# Physician autonomy and patient rights: lessons from an enforced blood transfusion and the role of patient blood management

**DOI:** 10.1111/vox.13106

**Published:** 2021-04-07

**Authors:** Matteo Bolcato, Aryeh Shander, James P. Isbister, Kevin M. Trentino, Marianna Russo, Daniele Rodriguez, Anna Aprile

**Affiliations:** ^1^ Department of Molecular Medicine, Legal Medicine University of Padua Padua Italy; ^2^ Department of Anesthesiology Critical Care Medicine Pain Management and Hyperbaric Medicine Team Health Research Institute Englewood Medical Center Englewood NJ USA; ^3^ School of Medicine The University of Sydney Sydney NSW Australia; ^4^ Medical School The University of Western Australia Perth WA Australia

**Keywords:** ethics, Jehovah’s Witness, patient blood management, transfusion medicine

## Abstract

This article provides an ethical and medico‐legal analysis of ruling no. 465 of 30 May 2018 issued by the Court of Termini Imerese (Palermo) and confirmed on appeal on 11 November 2020, which, in the absence of similar historical precedents in Europe, convicted a medical doctor of a crime of violent assault for having ordered the administration of a blood transfusion to a patient specifically declining blood transfusion on religious grounds. We analyse the Court’s decision regarding the identification of assault in performing the blood transfusion and its decision not to accept exculpatory urgent ‘necessity’ as a defence. In addition, we present an updated revision of the current standard of care in transfusion medicine as well as the ethical principles governing the patient's declining of transfusion. In doing so, we highlight that respect for the patient's self‐determination in declining transfusions and respect for the professional autonomy of the doctor protecting the safety and life of the patient could be equally satisfied by applying the current peer‐reviewed evidence.

## Introduction

A frequently discussed topic in clinical legal medicine is the right of patients who have mental capacity or have an advanced healthcare directive. Choice of specific medical interventions can be either accepted or declined by patients. The choice of declining blood transfusions by Jehovah’s Witness patients is a context for such discussion. In recent decades, in many parts of the world, declining transfusion has been the focus of important reflections on the rights of patients and a frustrating event for legal and medical professionals. For the latter, it seemed impossible to reconcile these patients' rights for self‐determination and the ethical purpose of medical practice to provide the best care to safeguard the patient’s life and safety. Numerous published examples of such cases are in the literature [1, 2]. However, there are no cases in Europe that have been detailed in the scientific literature in which the administration of a blood transfusion has been considered as an assault on a patient perpetrated by health professionals. In this article, we discuss a Court’s decision in Italy to convict a medical practitioner of criminal assault for administering a blood transfusion to a patient who specifically declined transfusion under any circumstance. The court did not accept exculpatory urgent ‘necessity’ as a defence. The case is analysed, and an updated review into the current standards of care in transfusion medicine and patient blood management is presented. This landmark ruling received significant media coverage in Italy in 2018 and 2020 and has broader relevance for clinical medicine.

## The case report

**Fig. 1:**
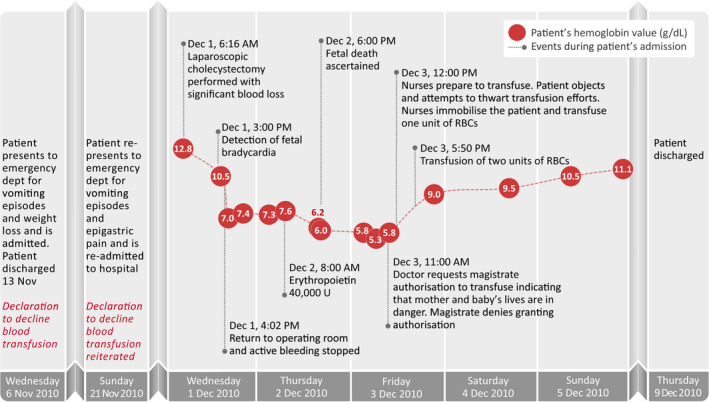
Summary of key hospitalization events.

On 6 November 2010, a 24‐year‐old woman was admitted to the emergency department of the ‘Cimino’ Hospital in Termini Imerese (Palermo, Sicily). The patient was in her 14th week of gestation and from the beginning of the third month of pregnancy experienced vomiting episodes that were difficult to manage resulting in 3–4 kg of weight loss. Her medical history comprises a previous term pregnancy requiring Caesarean section as well as a history of episodes of tachycardia. The young woman was hospitalized for further management, during which she declared herself as a Jehovah’s Witness. She expressed her intention to decline blood transfusion of any blood components (red cells, platelets and plasma), while accepting all patient blood management strategies aimed at the management of her own blood including the administration of blood derivatives such as factor concentrates. On 13 November 2010, the woman was discharged with the recommendation to take folate and supplements containing magnesium and potassium; the state of health of the fetus was satisfactory. On 21 November 2010, the patient was once again admitted to the emergency department of the ‘Cimino’ Hospital for epigastric pain and vomiting, followed by admission to the Gynecology and Obstetrics department. She again confirmed to be a Jehovah’s Witness and reiterated her refusal of any transfusion of blood components. Investigations during hospitalization included an ultrasound examination showing the gallbladder contained abundant biliary sand and micro‐gallstones. The patient was initially treated conservatively, but in the following days, symptoms of pain and vomiting reappeared and on 28 November 2010 the onset of hyperbilirubinaemia were observed. The clinicians determined the need to perform a cholecystectomy. Blood tests were performed, showing a haemoglobin level of 12.8 g/dl. On the morning of 1 December 2010, laparoscopic surgery was performed. The medical record does not report the time of start and end of surgery; however, the findings included in the medical record show that shortly after the end of surgery, a significant blood loss occurs both from the drains and from the surgical access points. Despite this, laboratory checks and gynaecological examination were not performed until several hours later. A gynaecological consultation was carried out at 3:00 p.m. and revealed fetal bradycardia (83 bpm). Atropine was prescribed and administered; a blood count and a reassessment after one hour were scheduled. Around 4:00 p.m., the patient was hypotensive and reoperation revealed active bleeding at the original surgical incision site, which was controlled via affixing parietal stitches and there was no further blood loss. The following morning, erythropoietin 40 000 U and intravenous iron 1 g/day were administered. The gynaecological consultation around 6:00 p.m. confirmed fetal death. On the morning of 3 December 2010, the haemoglobin level was 5.3 g/dl. A subsequent haemoglobin three hours later was 5.8 g/dl. However, 30 min earlier at 11:00 a.m. the medical record documented that, in light of the haemoglobin of 5.3 g/dl and in view of the patient’s religious commitment to decline blood transfusion, the physician considered informing the magistrate on duty at the Court of Termini Imerese, in order to proceed with an emergency blood transfusion. At 12:00 p.m., two nurses, following a direct order from the medical director of the clinical unit, and after making the relatives leave the hospital room, removed the ongoing infusion from the venous access on the upper right limb and attached a unit of packed red blood cells. The patient immediately demanded an explanation from the nurses involved, and in response, she was told that the blood transfusion had been authorized by the magistrate and she must not object and resist. The patient strongly expressed her dissent and attempted to thwart the actions of the nurses by moving her limbs; intervening staff forcibly immobilized her, and after some time, the patient desisted from trying to free herself. The staff made it clear not to think about removing the infusion apparatus in that ‘there would have been serious consequences’. The nurse coordinator, present during the transfusion that was administered by another nurse, later stated during the trial that ‘the woman was very sad and cried’. Subsequently, the patient was yet again transfused with two further units of packed red blood cells. (Fig. 1 presents a summary of key hospitalization events.)

It should be noted that the medical director reported to the patient that he would transfuse her because he had received authorization from the magistrate by telephone. The physician had requested the authorization from the magistrate, emphasizing that the life of the woman and of the fetus were in danger. However in actual fact, at the time of the call, the fetus had already died. The magistrate subsequently reported that he had been contacted by telephone about the matter but denied granting any authorization. On 7 December 2010, a labour induction abortion was performed, and on 9 December 2010, the patient was discharged home.

The patient subsequently filed a complaint, and the Public Prosecutor investigated the clinicians who took part in the various events already described. The investigation included the potential crime of culpable abortion relating to incorrect surgical procedures that seemingly caused the death of the fetus and the crime of assault pursuant to Art. 610 of the Italian criminal code for having voluntarily forced the patient to undergo the blood transfusion that she had repeatedly and ‘stubbornly’ (as cited in the medical record) refused.

## The legal ruling

At the end of a long trial, ruling no. 465 of 30 May 2018 was delivered, absolving the defendants of the offence of culpable abortion but convicting the medical director of the clinical unit for criminal assault by having ordered the blood transfusion that was declined by the patient. This ruling was later confirmed on appeal on 11 November 2020. In this publication, the focus is on this latter crime and not issues relating to the fetal death. With reference to the crime of assault, the ruling is of specific interest as it represents a legal precedent in European jurisdictions, in which coercion to undergo a blood transfusion in spite of the dissent expressed by a competent adult patient satisfies the legal definition of a crime of assault in criminal terms.

The ruling provides a comprehensive summary for the legal basis and legitimization of the medical and surgical activity in the Italian legal system, as well as of the issue of informed consent, recalling the most significant Court rulings on the subject, issued in Europe.

## Informed consent as the legal basis for medical and surgical activity

The current ruling cites and acknowledges the conclusions of a previous case of the Court of Cassation, Italy’s highest judicial body. They reported that consent given by a patient is an actual assumption of lawfulness of the activity performed by the doctor who administers the treatment, to whom a general right to treat irrespective of the will of the patient cannot be ascribed. In this regard, it maintains the principle of self‐determination; the will of the patient is the ultimate boundary (inalienable and enduring) of the exercise of medical practice. Indeed, the criterion defining and dictating the doctor–patient relationship is that of the free availability of the benefit of health for the patient in possession of his/her intellectual and decisional capabilities, according to a full autonomy of choices. This can also entail the loss of life that must always be respected by healthcare professionals. Therefore, the court concluded every individual has the right to choose between the ‘salvation of the body and the salvation of the soul’.

The Ruling of the Court of Termini Imerese also refers to various international sources such as the Convention on the Rights of the Child, signed in New York on 20 November 1989; the Convention on Human Rights and Biomedicine, signed in Oviedo on 4 April 1997; and the Charter of Fundamental Rights of the European Union, proclaimed in Nice on 7 December 2000.

## Blood transfusion in the absence of consent and necessity

The ruling of the Court of Termini Imerese convicted the doctor for the crime of assault and determined not to accept the argument of the defendant who, in his defence, invoked having acted out of necessity. In Italian law, the defendant can claim exemption from conviction if the medical intervention was performed in order to protect others from the imminent risk of serious personal injury. In this case, neither the presence of risk factors nor inadequate physiological compensation for anaemia or abnormalities in the patient’s vital signs was documented in the medical record. It is indicated in the ruling that, although the laboratory findings revealed a low haemoglobin value (5.8 g/dl at the time of transfusion, after a *nadir* of 5.3 g/dl), the patient was not in a life‐threatening condition. The court ruling includes clinical and laboratory data from which it may be inferred that physiological compensatory mechanisms for anaemia were present and were responding adequately for the reduced haemoglobin levels. To this end, the expert witnesses for the plaintiff argued that haemoglobin results ‘cannot constitute the sole parameter to be considered in the decision to carry out a transfusion, nor can it become an irrational element of psychological terror in clinical decision‐making’. In addition, case reports of patients, from the international scientific literature, with particularly low haemoglobin values but displaying adequate compensation were presented [3, 4].

The Court determined that the patient was not in a life‐threatening emergency state; however, even if the patient’s anaemia had deteriorated to engender the presumption of imminent death only avertable with transfusion, it would still not have been possible to deem exculpatory necessity applicable in pursuance to Art. 54 of the Criminal Code. The patient, fully aware of the possible repercussions of her decision, had expressly and repeatedly denied her consent to the blood transfusion.

The ruling indicates that ‘indeed, no so‐called compulsory emergency aid is provided for in our legal system, able to extend beyond the contrary intention of the subject concerned, given that the limit of exculpatory necessity, in the light of the above‐mentioned constitutional principles, is strictly limited to the premise whereby the patient is unable ‐ due to his/her condition – to lend his/her dissent or consent… the doctor cannot, therefore, impose the health treatment that s/he deems life‐saving upon any patient who knowingly and lucidly refuses it’.

This stresses that the only case in which it is possible to consider exculpatory necessity as a valid defence is that in which the patient is in a situation of incapacity of manifesting his/her will and has not previously expressed any preferences regarding the clinical picture entailing imminent and present risk of serious personal injury. The same indication is also acknowledged by the new law that in Italy regulates the consent to the medical act and the anticipated treatment provisions of 22 December 2017 no. 219, in article 1, paragraph 7, stating that ‘in an emergency or in emergency situations, the doctor and the members of the health team ensure the necessary treatment, in compliance with the wishes of the patient should the latter’s clinical conditions and circumstances allow for their implementation’.

## Unwanted blood transfusion and grounds of the crime of assault

For the crime of assault to exist, the ruling indicates two key elements must be clearly identified: (1) violent conduct; and (2) the event, namely what the person is forced to suffer against his/her will. In this case, the violent conduct first materialized in all the manoeuvres to introduce the peripheral venous catheter into the vein and thus inside the patient’s body. The event was implemented via the introduction of blood inside the patient’s body and via the haemotransfusion. The doctor was not faced with an unexpected emergency; on the contrary, he planned the transfusion well in advance despite the patient's repeated denial.

The ruling identifies the requisite of violence as any suitable means to quash the freedom of determination and action of the injured party and that the interest protected that describes the crime is moral freedom, to be understood as the freedom of spontaneous self‐determination. The law in question protects the psychological freedom of the individual and represses coercion, explicable in myriad forms used to exert pressure on the will of others, preventing their free choice. In the crime in question, the transfusion, combined with all associated preparatory activities, is envisaged as an act of violence against the patient refusing it.

## Aspects of a bioethical nature

The Court of Termini Imerese reiterates and determines the legal principle based on the personalist conception of humans: the will of the patient as the ultimate limit of the exercise of medical activity, and more broadly health care, in which the criterion governing the doctor–patient relationship, as well as the healthcare professional–patient relationship, coincides with the free availability of the benefit of health for the patient in possession of his/her intellectual and cognitive capabilities, according to a freedom of choice that can imply the sacrifice of life itself and that must always be respected by health professionals. The Court also underlines that ‘necessity’, if present, cannot be used as a ‘strategy for undermining the rights of every person’ [5].

The clinical case presented contains important teachings and reflections useful to the entire scientific and legal community. From a moral and ethical point of view, the doctor has acted on the basis of the principle of his own autonomy in making decisions in terms of appropriateness and usefulness of the health treatments to be performed on the patient. However, this principle did not take into account the limits imposed by the respect, no less important, of the patient's self‐determination. The doctor is necessarily autonomous as regards the strictly technical‐scientific field of patient care and management but cannot ignore considerations relating to the patient's lifestyle, values, needs and aspirations, who exercises his own autonomy in health choices.

In the specific case, it must be considered that the doctor acted on the basis of an inappropriate technical‐scientific autonomy, as the blood transfusions he imposed on the patient were not based on scientific evidence and therefore were in no way justifiable by failing to comply with the rules that define autonomy. This word is made up of two ancient Greek terms, αυτός and νόμος (which mean ‘own’ and ‘rule’), and expresses the competence to operate according to the ‘proper rules’ of the profession. According to a certain culture, still quite widespread, a paternalistic attitude towards the patient persists, according to which the doctor is the good father who knows the good of the patient‐children and acts accordingly.

The shift from a paternalistic doctor–patient relationship to one of shared decision‐making has been a slow and problematic one dating as far back to Hippocrates. The traditional view of this relationship became well imbedded for two millennia on the basis that a patient should be ‘protected’ from knowing the truth about their disease and its likely outcome. Hippocrates stated: ‘Reveal nothing of the patient’s present or future condition’. The rationale was that the fully informed patient may not be able to absorb, understand and psychologically cope with the information. Indeed, the meaning and origin of the word ‘patient’ exemplified the doctor/patient communication and interface. The word patient is from the Latin verb pati, to suffer. The words passive and passion have the same origin, and over time, the term patient has come to mean somebody who suffers their disease with calmness and composure and having patience. It was implied that appropriately informing the patient might cause stress or worse, harm the patient.

This paternalistic relationship between the doctor and patient was a modus operandi that excluded truth telling and informed patient consent. This almost implied the doctors’ service was a commodity that is assumed to be fit for purpose and ‘buyer beware’ (caveat emptor). The patient was expected to tacitly and unquestionably trust their doctor.

This type of approach is no longer tolerable in any way from an ethical point of view, in an evolved society where the person is at the centre of care and informed decisions about his life and his future, even up to the extreme consequences.

The paternalistic doctor distorts and mystifies the principle of charity [6], under the illusion that the patient's good may come from the doctor's wisdom and not from the evaluation of the interested party, who is free to avail himself of the support of others, without, however, others being able to arrogantly intrude on his decision‐making process or even, as in the case under discussion, to completely replace him. In this case, paternalism even led the doctor who carried out the transfusions not to apply the principle of non‐maleficence. In fact, through the transfusion he violated the intimacy (by creating a constraint) and the dignity (by disregarding religious beliefs) of the person; it then determined, as a consequence, a psychological insult and damage. To further clarify, it is worth considering the religious aspects of the case.

The religious component of identity also assumes importance, whenever the transfusion is offered to a person who practices a religion that prescribes rules of conduct that prohibit it. These are binding norms that do not lend themselves to personal re‐elaboration and that make the transfusion, if practiced, a permanent treatment that damages the religious identity of those who suffer it. In these cases, the irresolvable compromise of religious identity affects personal identity as a whole, since it is unthinkable that, in these irreversible circumstances, the person can be able to process the bodily aspects of his own identity compromised by the extraneous biological mass.

The compromise of religious identity, in addition to preventing the elaboration of bodily identity per se, intrinsically compromised [7], also has an impact on social and family identity: the first with reference to the social group of those who practice the same religion and the second with particular (but not exclusive) reference to family relationships when one or more members of the family belong to the same creed. In these cases, therefore, the transfusion carried out against consent leads to such a compromise of the identity, that is, involving different aspects of the same: religious, corporeal, family, social.

The doctor who practices transfusion, indifferent to these consequences, expresses lack of respect for the person as the bearer and expression of an intrinsic value and therefore also damages their dignity. If this lack of respect is public, because it is made known, with concrete acts, both to relatives and to those who accompany the person and follow the human story, the damage to dignity is also perceived and suffered by the family and social context.

## Patient blood management: a solution to manage the patient’s blood with better outcomes

Both the patient’s and the doctor’s best interests could have been satisfied by applying current peer‐reviewed evidence. In fact, the issue of minimising and avoiding blood in medicine and surgery is a topic of great interest, not only for patients who decline transfusions but also for the population at large [8, 9, 10].

For example, since 2002 the World Health Organization (WHO) has recommended ‘transfusion alternatives’ where possible to avoid exposing patients to the risks associated with blood transfusions [11]. More recently, in 2010, the World Health Assembly endorsed patient blood management (PBM) as the standard of care. PBM being defined as ‘an evidence‐based bundle of care to optimize medical and surgical patient outcomes by clinically managing and preserving a patient’s blood’ [12].

The principles and practical application of PBM initiatives first took place when assisting Jehovah’s Witness patients; however, the methods and techniques applied would later benefit all patients [13]. For example, in 2008 the Government of Western Australia successfully implemented a state‐wide PBM programme which led to significant reductions in transfusions and concurrent improvements in patient outcomes. As a result of the reduction in transfusions, tens of millions of dollars were saved [14]. In March 2017, the European Commission introduced a Guide intended to implement PBM as a standard of care throughout the European Union [15, 16].

Patients treated according to the principles of PBM have their own blood optimized prior to surgery and their blood loss minimized during surgery. With the application of these proactive approaches, the patient may not reach a restrictive transfusion threshold, minimising or eliminating the administration of blood products. Even when it is not possible to act prior to surgery, strategies for the management of postoperative anaemia after major surgery remain applicable, [17] and the literature has many examples of complex interventions performed without using blood transfusion, with results that are similar, if not better, than those of transfused patients [18, 19, 20, 21, 22, 23, 24, 25, 26, 27, 28, 29, 30, 31].

Patient blood management is not a specific medical intervention or an alternative to allogeneic blood transfusion; it is sound evidence‐based clinical practice. Minimising and avoiding blood transfusion is a corollary stemming from successful PBM. The principles of PBM are based on a robust understanding of core physiological and pathophysiological aspects of haemopoiesis, haemostasis and oxygen transport. The haematological and immune systems are the fundamental physiological infrastructure to maintain the body’s homeostasis, responses to injury and tissue repair. These systems have considerable adaptive reserve responding to deficiencies or increased demands. It is the responsibility of all clinicians who have primary accountability for the quality and safety of a patient’s clinical management to ensure the patient’s blood is managed appropriately [32, 33, 34]. It is no longer acceptable or ethical to continue adopting a *laissez‐faire* approach to the assumed benefits and known risks of allogeneic blood transfusion. Blood transfusion can no longer be regarded as default therapy in the context of clinical uncertainty. Managing a patient’s own blood appropriately is now the clinical decision‐making focus, based on the three pillars of PBM [35, 36]. If after following the principles of PBM, evidence‐based medicine suggests an allogeneic blood transfusion is appropriate, the consent process will still require a doctor to discuss the risk and benefits as well as any possible alternatives [37]. Where alternatives are not available, or the risk/benefit equation is not clear, and the patient does not decline transfusion, evidence is needed that the patient’s ultimate clinical outcome is likely to be improved. Surrogate endpoints are necessary for many clinical interventions, but it is necessary that these immediately measurable surrogate endpoints causally correlate with better long‐term patient outcomes.

**Fig. 2:**
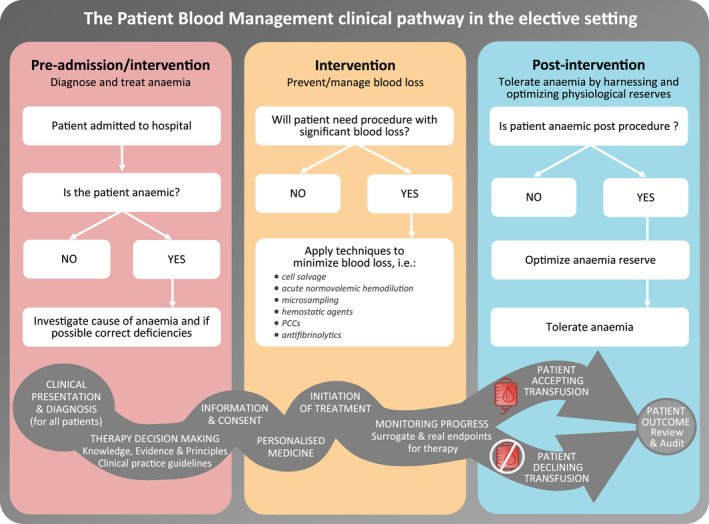
The patient blood management clinical pathway in the elective setting.

In circumstances in which a patient declines allogeneic blood transfusion, it is imperative that a pre‐emptive strategy is in place. This management strategy should be initiated and documented for such patients at the point in their clinical course that blood transfusion would normally be regarded as standard of care. An argument can be made that all patients should be managed on this basis up until blood transfusion is considered appropriate management (Fig. 2).

In the case reported, if PBM and the legal empowerment of the patient had been the *modus operandi* from the initial admission, the outcome for the patient and the doctor would have been quite different. To use current *lingua franca,* a lose–lose outcome could have been a win–win outcome.

## Conclusions

The ruling concerning the Termini Imerese case has roused much media interest in Italy. It is based on the fundamental principles of freedom and self‐determination universally acknowledged in the Western world for adults with mental capacity. The same situation, related to therapeutic choices in the case of potentially life‐saving therapies, can manifest itself in other situations, which are now regulated in Italy by law No. 219 of 2017 also referred to as the ‘Living will’ providing for the respect of the patient’s current will, as well as any anticipated treatment provisions, thus guaranteeing the right of self‐determination of the adult subject, possibly even expressing the declining of life‐saving treatments, in any clinical situation. In the case of declining transfusion, modern practice including PBM, when correctly applied, has greatly minimized the clinical problems and opened up a new perspective for the application of the principles of legal medicine in the field of medical professional liability.

## Funding

None.

## Conflict of interests

A. Shander has received consulting, research and lecture fees, as well as expenses, from Masimo, CSL Behring, IL Werfen, Merck, Vifor Pharma and HbO2 Therapeutics. Other authors declare that they have no conflicts of interest.
